# AprilTags in Unity: A Local Alternative to Shared Spatial Anchors for Synergistic Shared Space Applications Involving Extended Reality and the Internet of Things

**DOI:** 10.3390/s25144408

**Published:** 2025-07-15

**Authors:** Amitabh Mishra, Kevin Foster Carff

**Affiliations:** 1 Department of Cybersecurity and IT, HMCSE, University of West Florida, Pensacola, FL 32514, USA; 2 Department of Computer Science, HMCSE, University of West Florida, Pensacola, FL 32514, USA; kfc8@students.uwf.edu

**Keywords:** 4-point calibration, AprilTags, extended reality, internet of things, shared-space localization, unity

## Abstract

**Highlights:**

The highlights of this work are listed below.

**What are the main findings?**

- The serious privacy issue of the user being forced by extended reality (XR) headset manufacturing companies to have to upload the user’s setup and surroundings for traditional, legacy calibration can be addressed locally without compromising on the user’s privacy.
- By using Unity, Mirror Networking, and the QuestDisplayAccessDemo, a shared host system implemented locally can distribute location data with XR clients. Some performance analysis of the developed system is also included in this article.

**What is the implication of the main finding?**

- This is a no-to-low-cost solution for XR headset calibration and localization that circumvents legacy calibration systems for XR headsets that essentially need high bandwidth.
- The solution is completely local and does not need internet connectivity, unlike the critical and traditional initial setup that can be implemented only in places that have high-bandwidth internet connectivity. This means that expensive XR headsets can now be used in any user environment without needing internet connection for setting them up.

**Abstract:**

Creating shared spaces is a key part of making extended reality (XR) and Internet of Things (IoT) technology more interactive and collaborative. Currently, one system which stands out in achieving this end commercially involves spatial anchors. Due to the cloud-based nature of these anchors, they can introduce connectivity and privacy issues for projects which need to be isolated from the internet. This research attempts to explore and create a different approach that does not require internet connectivity. This work involves the creation of an AprilTags-based calibration system as a local solution for creating shared XR spaces and investigates its performance. AprilTags are simple, scannable markers that, through computer vision algorithms, can help XR devices figure out position and rotation in a three-dimensional space. This implies that multiple users can be in the same virtual space and in the real-world space at the same time, easily. Our tests in XR showed that this method is accurate and works well for synchronizing multiple users. This approach could make shared XR experiences faster, more private, and easier to use without depending on cloud-based calibration systems.

## 1. Introduction

A promising method for interactively visualizing design, construction, learning, recreation, and facility management is “extended reality” (XR), which has advanced recently. The term XR refers to the general idea of expanding or changing our reality through technological means to produce realistic and engaging settings and exchanges. XR is a blanket term used to refer to three technologies—augmented reality (AR), virtual reality (VR), and mixed reality (MR)—that aim to provide immersive and interactive experiences that combine the real and digital worlds. These technologies replace the user’s actual surroundings with a virtual one, creating a completely realistic virtual space for immersive experiences and interactions in real time.

### 1.1. The Development of XR Technologies and the Internet of Things

Originally, virtual reality was an artificial setting created with software to produce games and three-dimensional (3D) films that were as realistic as possible. Its extension, XR, is now an interactive environment in which the degree to which reality has been replicated on the program determines how fully a person is submerged in virtual reality. To create an immersive and participatory experience, a sensory experience is created, mostly employing sight and sound. XR changes how we perceive the environment and has the potential to completely transform cooperative relationships as technology develops and accessibility increases, changing how people communicate, collaborate, and consume immersive material as a group.

Typically, for XR, the main senses engaged in the encounter are sight and sound. A computer screen or a specialized virtual reality headset are the two ways that most modern virtual realities are shown. To produce the lifelike feelings that give XR its reputation as an immersive environment, more recent XR systems typically rely on XR headsets. For the user to concentrate on the virtual environment they are entering, most XR headsets come with a wraparound head-mounted display that blocks out light and real-world sights.

With the predicted proliferation of mobile AR devices and smart glasses in the upcoming years, the XR market is predicted to be worth over USD 98 billion by 2025 as its popularity continues to grow. It is expected to grow at a compound annual growth rate (CAGR) more than 32.7% to reach U.S. Dollars (USD) 3.06 trillion in sales by 2037 [1]. As technology develops, more appealing material, like video games, resources for learning, and other sources of recreation will start to emerge. Expanded gadget availability, consumer electronics implementation, entertainment and leisure usage, defense-related uses, and connectivity to the Internet of Things (IoT) are all factors contributing to the expansion [2]. The integration of spatial computing into extended reality applications and the expansion of generative Al in this context have also contributed to the growth of the market [3].

Providing the user with a suitably precise real-time location is crucial when establishing a location-based service, such as when creating virtual worlds for XR. Indoor localization can be used for a wide range of purposes, from guiding visitors or managing disasters around a facility to intelligently inspecting and maintaining building equipment or automatically deploying unmanned vehicles like robots. Satellite-based navigation systems are typically used for positioning outside. It is quite difficult to automatically place humans in satellite signal-free situations, particularly inside buildings as building elements like walls and ceilings deflect or attenuate satellite signals. Numerous methods for indoor localization based on different technologies, such as radio waves, ultrasound, and infrared, are being investigated to close this gap. Geo-localization has many practical applications, including 3D reconstruction [4], road maintenance [5], self-driving cars [5,6], robotics [6,7], and augmented reality [8], and it presents a variety of technological issues in computer vision.

The Internet of Things (IoT) is a network of linked electronic devices intended to simplify consumers’ lives in a vast technological environment. In recent years, IoT and XR (IoT-XR), which serve a wide range of different purposes, have joined forces to advance technology even further than before.

### 1.2. XR Applications

People can virtually participate in social gatherings through collaborative extended reality, which fosters a sense of connection and presence even when they are geographically apart. Teams working on creative projects can collaborate more effectively using shared extended reality, which enables in-the-moment communication and ideation and lets makers create on-demand animated spectacles with profound and enduring impact on users by fusing together several media. Possible applications involve 360-degree drone helicopter views, business presentations, and motion pictures that engage audiences through interaction. Localization is one of the major calibration challenges that are faced by all kinds of XR applications.

XR can be used by educational establishments to develop dynamic and captivating learning experiences that help students understand, learn, enjoy, and retain content from difficult subjects. XR can be used to offer more practical business training and instruct tradesmen, artisans, and even medical specialists like surgeons. Collaborative extended reality enables healthcare providers to undergo realistic training tasks and simulations that improve their medical capabilities and decision-making. Combining XR with apps that support mindfulness, relaxation, exercise, and meditation can result in a more immersive wellness experience. COVID-19 lockdowns brought forward the need for virtual conferences and meetings which can be facilitated by XR. Multiplayer XR can effectively connect distant people into proximity in an increasingly engaging and participatory setting.

As it allows players to fully immerse themselves in the environment of the game, XR is particularly popular in the gaming and amusement industry. Tourism promoters can now be able to digitally introduce someone to their most exciting sites, giving them the impression that the users are in that city. Virtual journeys and tour excursions through shared XR let people discover new places in the comfort of their own homes. Immersive XR technology enables the installation of 360° geodesic dome structures to simulate virtual tours in museums and create planetariums and cultural shows.

Establishing a shared comprehension of a project among important architecture, engineering, and construction (AEC) sector players who are spread out geographically is challenging. Real estate designers and architects can use XR for presenting their architectural design work and its visualization in an interactive way that helps clients more effectively comprehend and envision the designs. Compared to individuals who used a single-person VR system, XR users could succeed better in an engineering assessment task thanks to interpersonal interactions in the immersive virtual environment and communications in construction projects.

### 1.3. Applications of Synergistic IoT-XR

While XR has the potential to provide a more immersive way to view and interact with data, IoT devices can send this data in real time into XR worlds. Compared to traditional methods like dashboards, XR can increase the user’s feeling of immersion and presence in the virtual environment by integrating with IoT devices and providing a more engaging and interactive way to analyze IoT data. The sense of co-existence might be enhanced by a virtual avatar that interacts with real-world objects, such as turning on lights. IoT data-driven XR simulations can offer realistic training scenarios with real-time data such as physiological data, motion, and environmental conditions for a variety of occupations.

Complex data sets from IoT devices can be visualized in 3D using XR, which helps users comprehend and analyze the data. Three-dimensional visualizations in mixed reality created by IoT real-time databases [9] enable decision-makers to comprehend and extract insights from vast datasets. Some initiatives operate at a higher level than the IoT platform and are geared toward data integration [10], analytics [11], and applications [12].

#### 1.3.1. Medical Applications of IoT-XR

Utilizing IoT devices for data collection and feedback, XR may be used for distant monitoring of patients, patient rehabilitation, surgical training, and to keep track of patient health. IoT-enabled health tracking could involve XR-based exercise regimens that also track and record users’ activities using physical inputs like heart rate, movement, and body temperature. With health-focused biometrics, people and their doctors can now monitor and predict illness and other health problems more effectively.

#### 1.3.2. IoT-XR for Workplaces

IoT-connected devices may be explored in a virtual environment by engineers and designers, allowing them to evaluate their design and functionality prior to actual manufacturing. Even in remote work environments, everyone attending a meeting can make eye contact with the speaker because XR combined with IoT enables participants to seem like holograms in three-dimensional space. Businesses can improve their purchasing experience by setting up virtual showrooms where customers can interact with IoT-enabled items in a virtual setting.

Furthermore, by combining IoT data with machine learning (ML) algorithms, XR experiences may be tailored to the user’s preferences and actions. Robust collaboration can be produced, data visualization can be enhanced for a range of applications, and more credible and engrossing simulations with personalized experiences can be created because of this integration.

#### 1.3.3. IoT-XR in Smart Infrastructure

XR with IoT can be utilized to simulate and visualize intelligent city infrastructure, which improves decision-making and planning [13]. The synergy can be used for more efficient urban planning, administration, as well as for the construction of buildings [14], towns [15], and public infrastructure [16]. Further, it can be applied to environmental protection [17,18], too. A common data model for IoT devices facilitates consistent data interpretation and use across different platforms, apps, and smart city services.

#### 1.3.4. IoT-XR in Industry

The use of IoT sensors in XR can lead users through complex workplaces like factories or warehouses, delivering real-time directions and alerts. A “digital twin” is a virtual representation of an object, building, or system that is linked to its real-world counterpart via live data feeds. These replicas are employed in a wide range of uses, including systems prototype development, construction mapping, and security surveillance. Digital twins, when used in conjunction with the IoT and XR, allow individuals to make better financial choices as well as construct projects more effectively by identifying and resolving safety concerns before making significant financial commitments [19].

When paired with the IoT, XR enables remote monitoring and management of equipment in industrial or dangerous environments along with asset tracking, predictive maintenance, and remote monitoring. With IoT-XR, technicians can see a machine’s state, maintenance record, and repair directions overlaid on the device itself. Data visualization after optimization is essential in many industries for making sense of the vast amounts of data collected and determining the most effective and productive use of that data.

XR headsets can walk employees through intricate assembly procedures by superimposing step-by-step instructions over the real workspace. IoT sensors can monitor progress and quality in real time and make changes to instructions as needed. Potential quality issues can be identified before they arise in the actual manufacturing line, energy generation [20], and agriculture [21].

#### 1.3.5. IoT-XR in Learning and Entertainment

IoT data feeds into VR simulations, allowing employees to practice real-world training scenarios without any physical danger. Using real-time data from IoT devices an XR game may change its difficulty level in response to the user’s heart rate, which is monitored by a wearable IoT gadget.

An IoT-based XR pipeline can be used for online music education, where the real-time status of each student in an online class can be determined using graph neural networks [22]. The need for metadata for creating complete data models that enable interoperability is now the focus of standardization agencies and industry groups [23,24].

### 1.4. Shared XR

When it started, XR was limited to personal and isolated virtual experiences, but it is now possible to share the engrossing XR experience with others. Shared XR encourages communication, cooperation, and shared feelings, allowing team members to work together to solve problems and accomplish team tasks in a shared virtual world, explore far-off places, or work on virtual design projects. In shared XR people can be physically present in a 360° open environment while immersed in extended reality.

Shared XR allows use of the live broadcast and recorded live video replay in several locations within the same venue to let teams adapt plans in real time to anticipate and avoid or solve any problems.

Creating shared space experiences is a necessary advancement for technology used in XR systems for unique collaboration and interaction. However, existing solutions for shared spatial localization, such as spatial anchors, rely heavily on cloud-based infrastructures [25]. The need to share data with the cloud can present significant challenges for projects requiring offline or private operation. While these systems provide global accessibility, they introduce a dependency on internet connectivity and raise privacy concerns, particularly in scenarios where local solutions would suffice.

### 1.5. XR Headsets and Domes

The XR headset contains all electronics with a screen. There is a mobile processor with wireless capabilities for computations. There are no cables and accessories, so the user has a lot more freedom to move around in XR, which enriches the XR experience. A chip in the XR headset controller calculates the interval between the pulse and the hit of the laser’s sweep on every axis. This makes it possible to calculate a position in a large space with high accuracy. An XR headset can convert a user’s actions in the actual world into comparable movements in the virtual world by precisely locating the helmet-mounted display (HMD), controllers, and other peripherals and determining how each is positioned.

XR must instantly mirror the motion a user makes in the real world as humans are sensitive to latency. For the perceived quality to be adequate, the tracking precision must be submillimeter. To function in any place under a variety of lighting and climate settings, the headset computation system must be resilient to a broad range of situations. Because the system is transportable and cannot be powered by cables, it must be computationally light and power efficient.

Only when XR devices connect to the cloud via a wired connection can they update their screens at the speed needed. The entire experience could be ruined by any delays or interruptions. With wireless headsets, this becomes difficult. Individual as well as shared XR can now be experienced through a sizable XR dome or projection cylinder that serves as the canvas for the shared experience in place of separate individual headgear.

### 1.6. XR Sensors and Calibration

Creating virtual sets and fusing simulated components with real-world video captures require precise tracking. This makes precise calibration of the XR devices very important. XR headsets, domes or projection cylinders are the common means used for multiple XR applications for providing users the virtual experience. These gadgets use several sensors, with cameras being the most important of these sensors.

Any sample manufacturing variation means that two sensors produced from the same manufacturer may yield slightly different readings. Sensors are subjected to varying environmental conditions during storage, shipping and/or during assembling and may show a change in response for the same input. Differences in sensor design and aging could also cause two different sensors to respond differently in similar conditions.

The solution to such issues is in sensor calibration which improves sensor measurement accuracy and adjusts for the reasons of variation in sensor response. The sensor-user provides a known stimulus to the sensor, records the measured value, and makes some adjustments to map this value to the expected value. This is performed for a range of values to ensure accurate output values from the sensor.

## 2. Literature Survey

Location is an important aspect of background that people unconsciously consider when deciphering a scene’s significance. Pictures can be used to geo-localize items. Extracting position information from pixel data in photos is possible as research suggests. The set of global positioning system (GPS) coordinates that an image or items inside an image are projected to belong to is specified by visual geo-localization techniques. Creating a map of the surrounding area, anticipating the scene in an image, or comparing the image to other comparable photographs from a database are some other suggested techniques. Various camera views, such as aerial, hybrid, or ground, could be used to capture the source photos. While a video geo-localized in a rural area might be helpful to those conducting land surveying, identifying that a picture is in a renowned tourist destination means that the image contains well-known attractions.

### 2.1. The Challenge of Localization

Predicting the relative position of objects or images for XR use within a comparatively small-scale, localized context is the fundamental objective that all visual localization techniques share. The goal of camera pose estimation, a crucial subfield of visual localization, is to forecast the camera’s orientation and location in relation to an object or location in a picture.

Visual geo-localization finds an object or image’s geospatial location on a global scale. The algorithm’s objective is to forecast the GPS coordinates of each image or item, since input photographs may come from anywhere in the world. Localization techniques based on computer vision have expanded into interconnected subfields such as visual localization techniques [26,27] or visual geo-localization techniques [28,29,30,31]. Visual localization techniques are used on a lower spatial scale and anticipate the locations of objects or images within a predefined spatial representation in a limited area [26,32].

Since the camera attitude is usually changing while the scene or object is fixed, it is necessary to infer the camera pose from each image in relation to the scene being shown. The camera pose is different from the camera position. The former includes the rotation of the camera’s *x*, *y*, and *z*-axes, while the latter only describes the location of a camera in a local coordinate system. While image localization algorithms just predict camera location, camera posture techniques anticipate the entire camera pose.

The problem of estimating the location of an image based solely on visual information is difficult because the photographs may have been shot under different settings. Images still include helpful context information that might aid with geolocation. Finding an image’s geographic position from just one camera or view is the goal of single-view image geo-localization. Nowadays, image retrieval is frequently used as a stage in pipelines for cross-view picture geo-localization [33,34]. Gu [35] carried out a thorough investigation with a particular emphasis on satellite photography.

The locating service sector has grown rapidly because of the quick development of mobile internet technologies. The deployment of base stations [36] and satellites [37] can only satisfy the needs of people in an outside setting. However, the need for indoor placement is becoming more pressing as more people in modern life are found in crowded areas like railroads, airports, indoor shopping malls, and work units [38].

The most widely used indoor positioning technique, which has greatly benefited people’s daily lives, jobs, and studies, is taking pictures and gathering them using cellphones to create indoor maps [39,40,41]. Unfortunately, the arrangement of each building floor is not very distinct, and some logos may appear more than once on different floors, particularly in major retail malls or hospitals. This makes it difficult to locate huge targets using two-dimensional placement methods [42]. Indoor Floor Localization (IFL) can use barometer data and Wi-Fi signals [43,44], which have the drawbacks of being prone to environmental fluctuations, requiring a lot of equipment, and being difficult to acquire. Due to their distinctiveness, geomagnetic signals can be used as an indicator for indoor finding [45].

Indoors, building elements like walls and ceilings can attenuate, shield, or reflect satellite signals. Based on various technologies like Wi-Fi, RFID, or UWB, autonomous indoor location is feasible for specific applications. A conventional solution is still unavailable, though. As widely used and reasonably priced multi-sensor devices, smartphones hold great promise as a mass-market indoor localization platform. An innovative approach based on the Sequential Monte Carlo method is described for the fusing of various linear and non-linear data [46] that could be available from smartphone sensors.

Indoor localization can be used for a wide range of purposes, from guiding visitors or managing disasters around a facility to intelligently inspecting and maintaining building equipment or automatically deploying unmanned vehicles like robots. Providing the user with a suitably precise real-time location is crucial when establishing a location-based service. However, because building elements like walls and ceilings deflect or weaken communications from satellites, it is currently unable to determine a position continuously and reliably inside buildings, even with specialized satellite navigation receivers [47,48].

‘Simultaneous localization and mapping’ (SLAM) is an approach to localization broadly seen in robotics [49]. SLAM algorithms create mapping of the immediate surroundings which, like all visual geolocation techniques, are local in scale. In SLAM, which is a computer vision method of localization, the starting point serves as the coordinate frame’s origin because there is neither a GPS nor an absolute coordinate frame for localization. The robot must use established landmarks in the surroundings, to probabilistically predict its route as it moves. In addition to calculating its own position in relation to the fixed markers, the robot also calculates the positions of the markers in relation to one another. Thus, both localizing against the map and estimating its position continue to occur concurrently. The headsets use SLAM for inside-out tracking [27,50,51,52,53]. In juxtaposition, a relatively simple computer vision algorithm such as AprilTag search is an elegant solution to the Shared-Space problem. While SLAM can localize a camera in a given space, it is computationally expensive for a relatively lightweight computing platform as the headset.

### 2.2. Cloud-Based Localization

Instead of depending on the processing capacity of the XR headset itself, cloud-based localization uses distant servers to interpret visual and inertial input from the headset so that more sophisticated tracking and localization methods can be used. Inertial measurement units (IMU) and cameras are used by VR headsets to record movement and visual data about the user and their environment. For processing, the IMU and visual data are sent to a cloud server for generating a map of the surroundings and estimating the headset’s orientation and position.

A cloud-based multiuser VR headset system that enables interpersonal project collaboration in an interactive VR environment is presented in the study by Du et al. [54]. A new AR headgear proposed in ref. [55] utilizes augmented reality, cloud, and eye-tracking capabilities, in a cloud-based headset to integrate wireless holographic display, a mouse and voice input. The authors claim that this allows users to handle their smartphones with just one finger rather than their hands. A new social VR-IoT environment is presented in [56], enabling users to collaborate on and manage nearby or distant IoT devices via a virtual platform. The VR-IoT solution is used in two different ways: one that is cloud-based and another that is local network-based. In ref. [57], authors have tried to address the challenging bitrate and latency requirements using lightweight VR glasses that wirelessly connect with edge/cloud computing devices that perform the rendering remotely.

The authors in article [58] suggest a cloudlet-based cloud computing system that allows Wi-Fi interior localization and navigating over a one-hop wireless network offering real-time interactive reaction data. To provide a headset tracking system, this research loosely ties a visual simultaneous localization and mapping algorithm to a closely connected carrier phase differential GNSS and inertial sensor subsystem [59].

Map-free Augmented Reality Localization (MARLoc) is a SLAM-like framework which claims to have reduced computational complexity [60]. This approach is novel in its ability to retain localization without maps. However, testing on specific VR devices is yet to be seen. Due to this, it is unclear how well the hardware would perform with this method.

### 2.3. Benefits of Cloud-Based XR Sensor Calibration for Localization

Sophisticated positioning techniques need greater processing power available with cloud servers. Extended reality headsets can be made smaller and lightweight by outsourcing computing to the cloud. Larger settings and more users can be accommodated by scaling cloud-based solutions. Certain headsets track position and orientation using SLAM and cameras that are installed on the headset. The virtual world is then rendered using data processing carried out on a cloud server for more accurate and reliable tracking.

### 2.4. Limitations of Cloud-Based XR Sensor Calibration Addressed in Present Work

Spatial anchors [61] offer global accessibility to shared localization. However, they are cloud-based and depend on internet connectivity. This introduces potential privacy issues because spatial data is being set to non-local servers [25]. Although cloud-based localization tools offer numerous benefits, they have a few drawbacks and restrictions.

To access and utilize cloud-based localization platforms, a steady and quick internet connection is necessary. It could be difficult to access and use the platform if internet connectivity is spotty or unavailable. Most cloud-based platforms cannot be tailored to an application’s unique requirements. This severely restricts the platform’s adaptability. Businesses that use cloud-based platforms might not have full authority over the data saved in the cloud or the localization procedure. For groups with specific needs or limitations, this could be an issue. Moreover, sensitive data is stored on platforms that use the cloud, which presents security risks. Data leaks, illegal access, and cyberattacks could jeopardize the data’s security. So, when employing cloud-based technologies, some organizations could be worried about the protection of their data. Privacy problems may arise since the data is kept in the cloud and may be accessible to the cloud service provider. All the other research works discussed in Section 2.2 have these inherent limitations.

For companies with extensive localization projects, cloud-based platforms can be costly. As more data needs to be localized, the cost can go up, and businesses might have to pay for more bandwidth and storage. XR equipment manufacturers deliberately design their XR devices to allow only for cloud-based localization and calibration and keep their libraries private for reasons of intellectual property rights (IPRs). The library or its source code are not shared with users if they want to use the purchased XR equipment in a purely local setting. This not only unethically forces the users to use their XR equipment in a place with an internet connection for cloud access but also rules out the use of their equipment in a place with poor to no internet connection. If the XR equipment is not calibrated correctly, it is hardly of any use in virtual settings. The current work is an attempt to circumvent this serious limitation and drawback by offering a completely local calibration of XR equipment that does not need an internet connection.

### 2.5. AprilTags for Local Calibration

The current reliance on cloud-based systems for spatial anchors brings up a need for alternatives that offer local, efficient, and precise shared space localization without any cloud dependencies. The challenge lies in designing a system that maintains repeatable accuracy and easy synchronization for multiple users while being self-contained on a local network. The ability to deploy such a system in unique spaces, free from Internet connectivity, broadens their applicability in sensitive sectors.

AprilTags are scannable markers that use computer vision to determine the precise position and orientation of a tile in a 3D space [62,63]. They have been widely adopted in robotics for similar localization tasks due to their simplicity and robustness [64]. This paper explores the use of AprilTags as a local solution for initializing shared spatial environments. By integrating AprilTags into a Unity-based server-authoritative system [65], this project employs a VR localization method that eliminates the need for cloud infrastructure. It also offers a lightweight, inexpensive, and privacy-conscious alternative for shared spatial localization.

## 3. System Design

**Figure 1:**
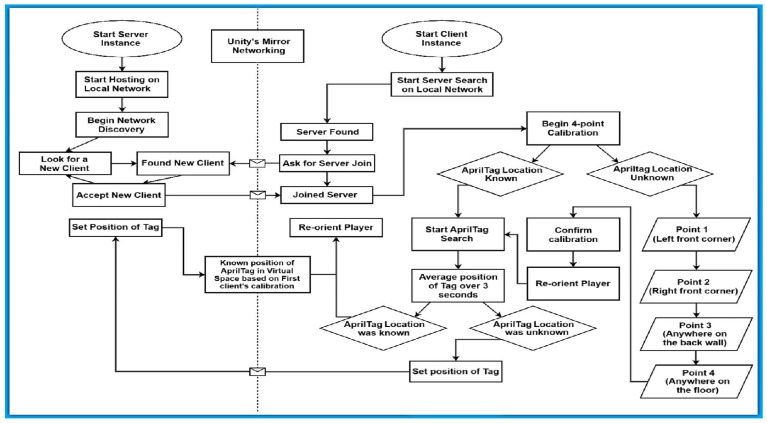
Flow chart of client–server communication during localization process.

The following section describes Figure 1 in detail so as to represent the system design and methodology of each general part of the system.

### 3.1. Server Side

The server instance acts as an authoritative dedicated server. Its purpose for this application is to share data between the clients as a mediator. The server instance utilizes a built-in VR Simulator. Upon starting, the VR Simulator’s player can be manipulated to click a virtual button called “Host.” This button will then start the network discovery process. It opens a User Datagram Protocol (UDP) port that broadcasts the server to the local network. When a client joins, it is given a player puppet so it can be visualized to other clients.

### 3.2. Client Side

To start with the client, a headset must own the Android Package Kit (APK) for the project which serves as a container for all the code, resources, assets, and manifest files required for an application to operate on a device. The APK supplies all the code, resources, and settings required to install an application on a device. In the headset hub, the client can then start the application. Upon start, the client does not immediately look for the server. To join the server, the client must click the “client” button. The client will then join the first discovered server in its network discovery.

### 3.3. 4-Point Calibration

Mapping the real-world coordinates to a virtual environment, such as a game engine or a virtual production stage, follows the four-point calibration process. Four-point calibration is a method used to determine the pinpointed position and orientation of a tracked device, like a camera, a robot arm, or any device whose position and orientation need to be determined—by using at least four physical points in the real world with known coordinates. This technique is commonly employed in fields like virtual production, where precise tracking is essential for creating realistic virtual environments. It involves mapping the real-world coordinates of these points to the virtual world, allowing for accurate alignment of virtual objects with the real-world camera’s view.

The four-point calibration method is a way which has been found to reliably localize a space. These points are placed by the headset by clicking the ‘a’ button on the right controller. These points are positioned at the coordinate position of the controller when the button is pressed. First, the client places a point in the front left corner of the room. Then, the front right corner-point is placed. These two points give the front wall of the room. The third point is placed anywhere on the back wall of the space. This third point gives the direction which the front wall faces. Finally, the fourth point is placed on the floor of the space. Headsets which employ inside-out tracking often lose track of the floor’s height between sessions. This fourth point gives a way to make sure the floor is where it needs to be for every calibration.

### 3.4. AprilTag-Based Localization

AprilTags have been widely used in robotics for their simplicity, robustness, and efficient pose estimation [62]. Their binary encoding makes them computationally lightweight, allowing for real-time tracking in various applications. A sort of optical fiducial indicator, AprilTags are extensively employed in robotics for computer vision tasks such as object size estimate, calibration of cameras, and navigation. AprilTags are 2D barcodes containing digital information that can be easily and precisely detected by camera-based sensors in applications that utilize computer vision. They allow for accurate localization and orientation within a scene or image by acting like identifiable optical markers. They are appropriate for calibrations of cameras, augmented reality, and robotics for assisting the equipment in understanding their location and orientation in the actual world. They are resilient to changes in the angle of light ray incidence, illumination, and variations in viewing angles. AprilTags are made to be easily identified and can be used even in scenarios with partial visibility. Hence, they are better suited for scenarios where exact orientation and position are essential since they focus on reliable detection. In essence, AprilTags are robust and can be recognized at greater distances since, in contrast to QR codes which are different in design and offer low overhead as they only encode a little quantity of data (4–12 bits v/s 3 KB for QR) even though they are both 2D barcodes.

XR cameras can be calibrated with AprilTags, which aid in identifying both the exterior and internal properties of the camera. Researchers can gauge the aberrations of the camera and calculate the necessary adjustments by knowing the orientation and position of AprilTags in the camera’s field of view. Proper calibration enables precise measurements and 3D picture reconstruction by cameras. On a field or in an environment, AprilTags are used to determine a robot’s orientation and position with relation to a predefined flag and thus assist robots in proactively navigating to designated areas on a field during robotic operations. Real-time item or robot movement tracking is also possible with AprilTags.

AprilTags are useful in determining the positions and ranges between an object and a camera. Tasks like aiming at a target, regulating a robot’s speed according to its distance from a target, or repositioning a virtual object in augmented reality can all benefit from this knowledge. AprilTags can be employed as moorings in augmented reality to superimpose simulated materials on the actual environment. A robot can use an AprilTag as a landmark to track its own position while completing a task.

In AR, AprilTags have been explored for marker-based tracking, as seen in Ultraleap’s research on AR Marker Tracking [66]. AprilTags’ effectiveness and deficiencies in mixed reality environments have also been demonstrated in studies like Illinois Mathematics and Science Academy (IMSA)’s analysis of object tracking with AprilTags [67]. Additionally, AprilTags have already been applied in VR for full-body tracking, as documented by Klug’s implementation in AprilTag-based motion tracking systems [68]. These examples demonstrate AprilTags potential as a lightweight, offline-friendly platform for localization.

### 3.5. Augmentations to the Original AprilTag Search

When the repo was first forked, the tag was, for lack of a better word, extremely jumpy. So, the first fix which was implemented was a slow linear interpolation called lerp() [69]. This function allows GameObjects to be given a position, and a timeframe to reach that position. This function is given three parameters which for ease of understanding will be referred to as Vector3 start, Vector3 end, and float time. A Vector3 is an object which consists of three floats *x*, *y*, and *z*. Often, these Vectors are used to discern an object’s position, Euler rotation, or scale. The start Vector3 parameter describes where the given object begins. The end Vector3 parameter describes where the object should end. The float time parameter describes how long in seconds it should take for the start position to reach the destination. The function lerp() outputs a Vector3 value in the form of start + (end − start) × time. Therefore, while repeatedly calling this lerp() function, a GameObject’s position might be set incrementally to smooth any rough pose estimations. While this fix improved the tags visually, the code changes did not entirely fix the issue of the tags jumping around. The tag seemed to oscillate in a seemingly harmonic motion. So, to fix the jumpiness, the average position and rotation of the tag was taken over the course of 3 s of staring at the tag. Moreover, the addition of lerp() was necessary due to the nature of data collection for correct position estimation. It was found that when aggregating point data, the large leaps which the estimated pose would make would drastically and often skew the mean position [70]. This fixed the jumpiness and allowed the aggregate to be used in the localization process to great effect.

## 4. Implementation

An application programming interface, or API, is a collection or repository of guidelines and conventions that facilitate data interchange and communication between various software programs. It is a contract or agreement between software components that outlines the process by which one application might ask another for data or services. It serves as a bridge, allowing apps to communicate and exchange features without having to understand the intricacies of one another’s internal systems. By enabling developers to incorporate pre-existing functionalities rather than creating them from the start, it streamlines the development process and allows developers to produce fresh and creative applications by giving them access to external data and services.

Our implementation builds upon the QuestDisplayAccessDemo repository [71]. This repository was foundational for the development of this project. It gave the basis for the AprilTag research which was performed. Through the android APK, the project allows the developer to analyze image data from the left-eye of the Quest Headset. The image’s dimensions are 1024 × 1024 pixels with an 80-degree field of view. While this image density is limited, it is workable. An issue with this method is that you can only obtain the image data as it is from the headset. So, the given image is not direct camera data, it is the screen data from the left eye only. Regardless of these limitations, the application programming interface (API) works well. Using the headset’s passthrough feature, the image data is very similar to the left eye’s direct camera data. Thus, when using this method, one must be sure there is not a virtual object obscuring view [72]. The following section describes the major components of the project, and how they were implemented.

### 4.1. Image Processing

**Figure 2:**
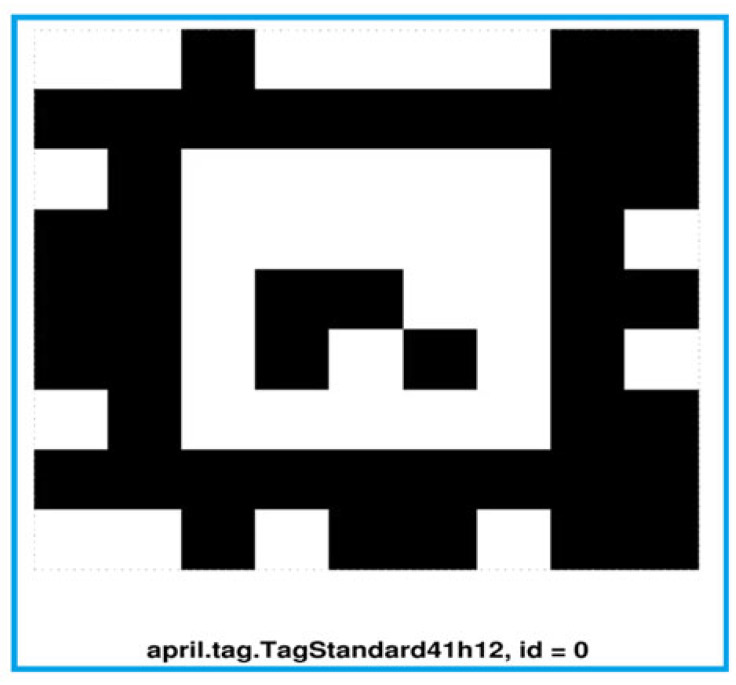
AprilTag used in the experiment.

The system accesses image data (1024 × 1024 resolution) from the headset’s left-eye passthrough. The AprilTag detection algorithm decimates the frame to speed up processing before scanning for the marker in Figure 2. In this project, a decimation factor of 2 was chosen for optimal performance in AprilTag detection.

**Figure 3:**
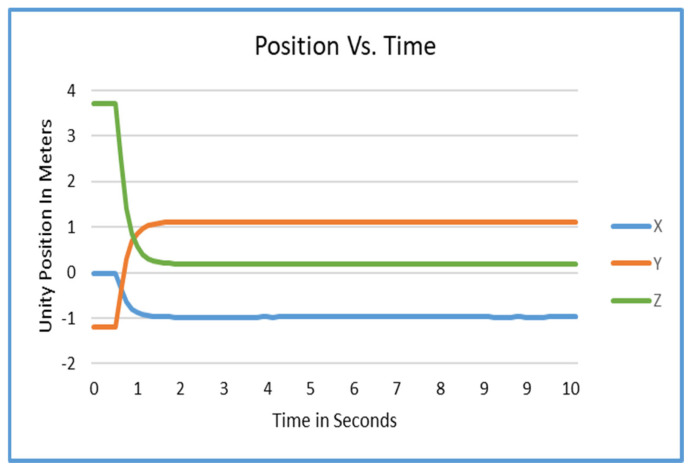
AprilTag positioning.

Figure 3 shows a graph of the position of the AprilTag over the course of 10 s. It was observed that on average it takes about 1.7 s for a tag to stabilize using the current method.

**Figure 4:**
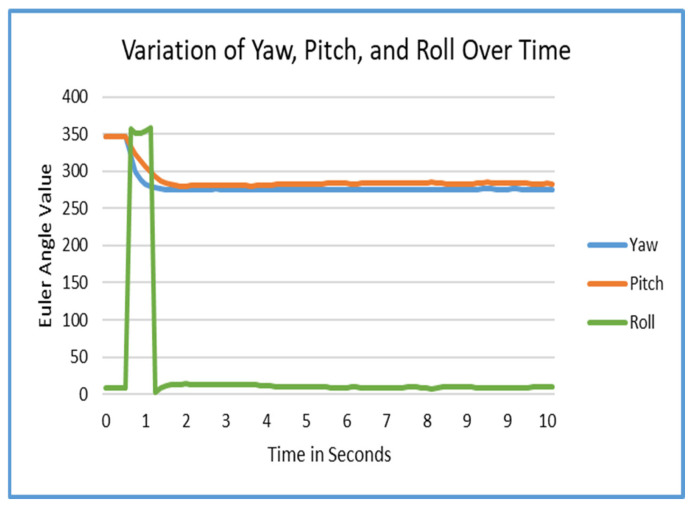
Roll, pitch, and yaw variations.

Figure 4 displays a graph of the rotation of the AprilTag over the course of 10 s. We found out that it takes about 1.7 s for a tag to stabilize using the current method on average.

### 4.2. Tag Smoothing

To counteract oscillation and “jumpiness” in the raw data, linear interpolation (lerp) and averaging over a three-second window was applied. Results for this can be seen in Figure 3. For the rotation of the tag, a simple averaging method reduces rotational error from up to ±2° to imperceptible levels under typical conditions. These results can be seen in Figure 4. As a future improvement, the tag’s convergence interval (1.7 s) could be significantly reduced. After the initial detection of the tag, the algorithm could instantly translate and rotate the tag to the initial estimated position and rotation by initially bypassing the lerp process. Then, the tag could be stabilized rapidly and easily exceed the 1.7 s stabilization minimum.

### 4.3. Network Communication

The first step involves the detection of the AprilTag. Post detection, the spatial data of the AprilTag is computed. Next, the information is transmitted from the client to the server over Mirror Networking protocols [73]. The server then relays the updated position and orientation on all clients. This is an important step that should be taken to ensure that all the headsets participating in the shared space are synchronized within the shared space.

### 4.4. Calibration Integration

A four-point calibration method is employed to calibrate the XR headsets. To begin the four-point calibration, four different locations in the physical world are selected to serve as calibration references. The tracking device is then moved along several orientations and locations, recording the four spots’ actual coordinates at each location.

To translate the real-world points to the virtual space chosen for mapping, the calibration process uses the recorded data to compute the transformation (translation and rotation) that correlates the real-world points with their matching virtual coordinates. By checking the monitored orientation and position of the device with the virtual environment, the final phase involves verification of the precise alignment of the virtual items with the real-world environment. Users mark the front left corner, front right corner, the back wall, and a floor point to guarantee accurate localization.

Not all the participating headsets need to complete this step. Only the first headset must calibrate their space using this method. This takes care of the calibration for the shared user set.

### 4.5. AprilTag Search

While scanning, the headset takes frames of the left eye. The search script then scans them in search of a tag. If a tag is found, the tag’s number is checked to see if there is a Unity GameObject which is looking for that number. If such a GameObject exists, the object’s transform is translated and rotated to match the perceived AprilTag position. In the absence of a tag, the process is reinitiated from the start.

### 4.6. Order of Operations

For this method of localization to work, a player must first calibrate their room. This is currently accomplished with the 4-point method mentioned above. Then, the player who calibrated must set the position of the AprilTag in their scene. This tag’s position and rotation are then communicated to the server. The server then distributes the knowledge to all other clients. After the location of the AprilTag is known, other clients can simply look at the tag to localize their own scenes. After all clients have been localized, they will be able to see each other’s avatars in virtual space overlapped with their bodies in real space.

## 5. Experimental Results

**Figure 5:**
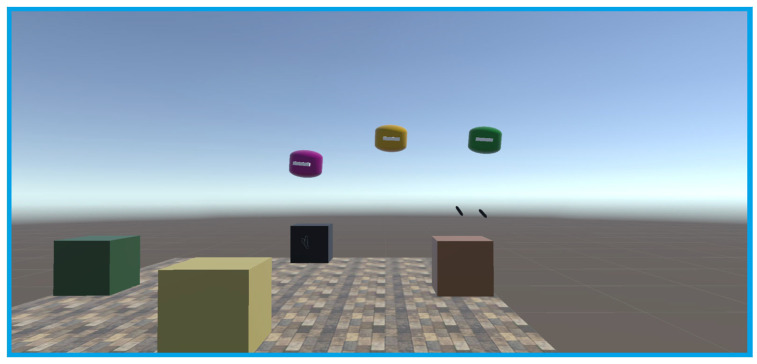
Forward view of Unity visualization for three-headset setup in a line.

**Figure 6:**
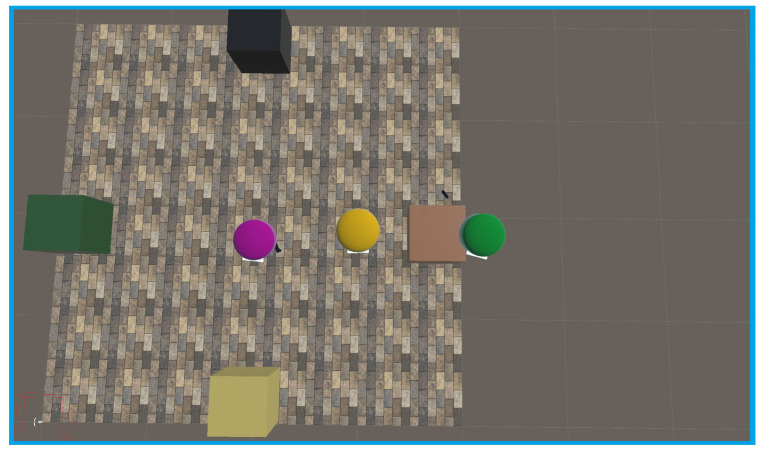
Top-down view of Unity visualization for three-headset setup in a line.

**Figure 7:**
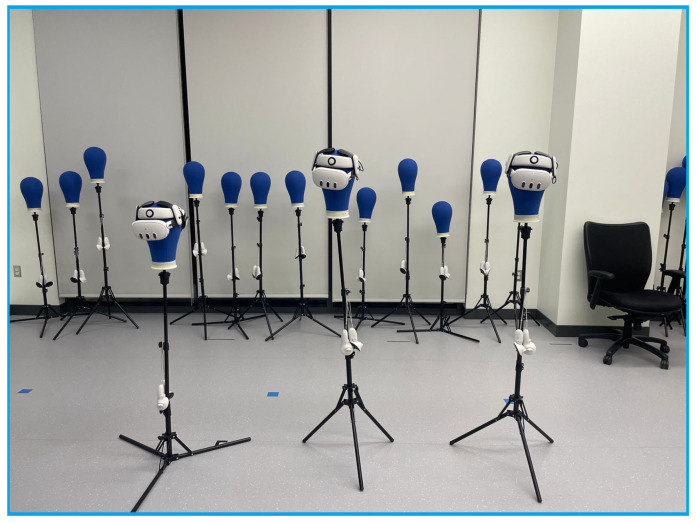
Three XR headsets in real space post calibration setup in a line.

**Figure 8:**
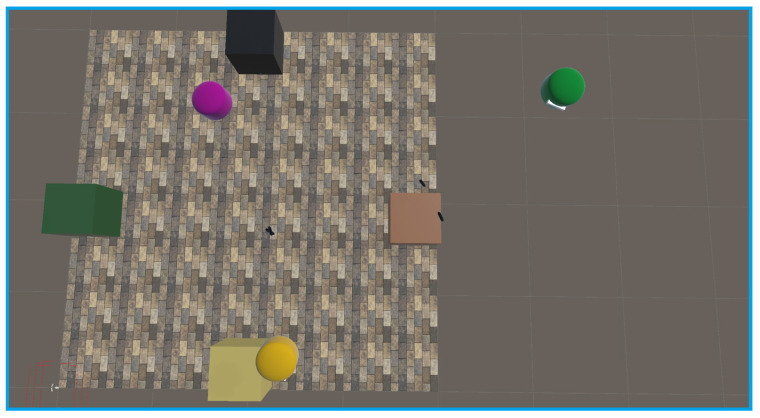
Top-down view of Unity visualization for three-headset in a spread-out configuration.

**Figure 9:**
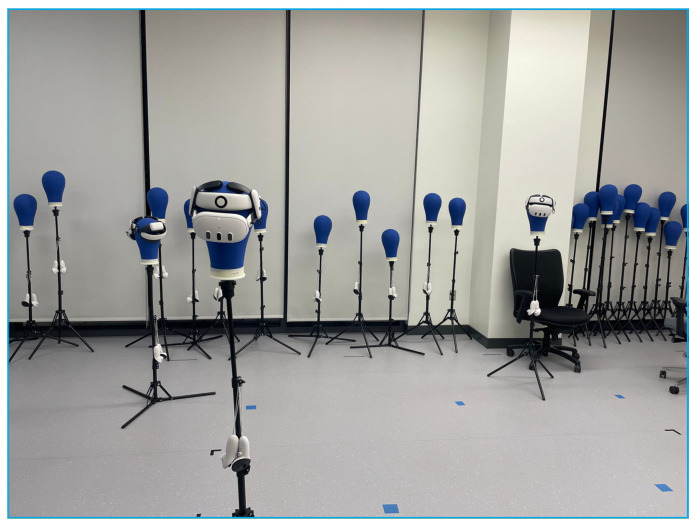
Three XR headsets in real space post calibration setup in a spread-out configuration.

**Figure 10:**
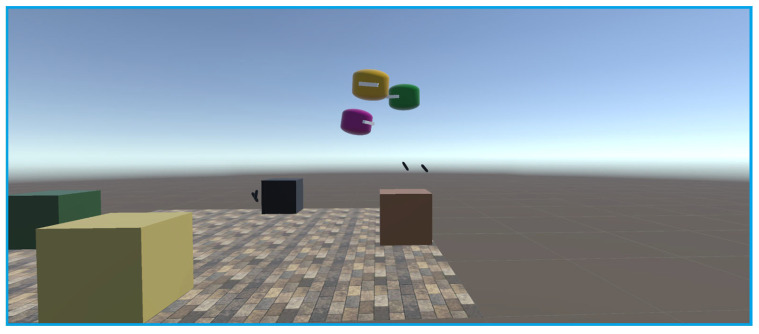
Forward view of Unity visualization for three-headset setup in a cluster.

**Figure 11:**
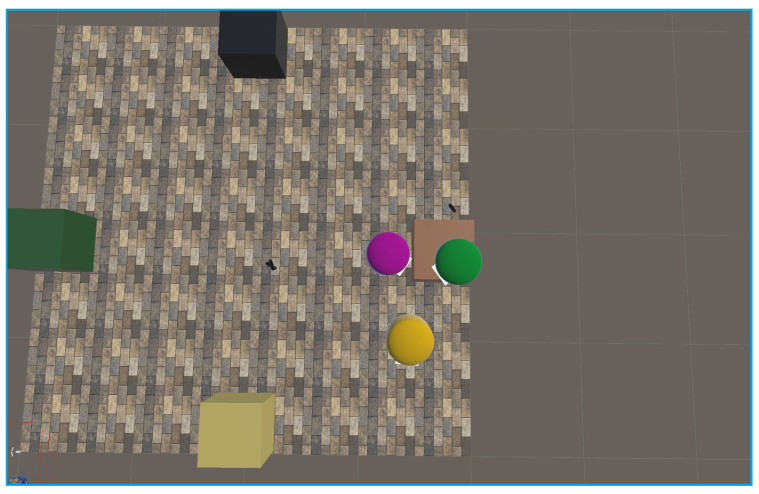
Top-down view of Unity visualization for three-headset setup in a cluster.

**Figure 12:**
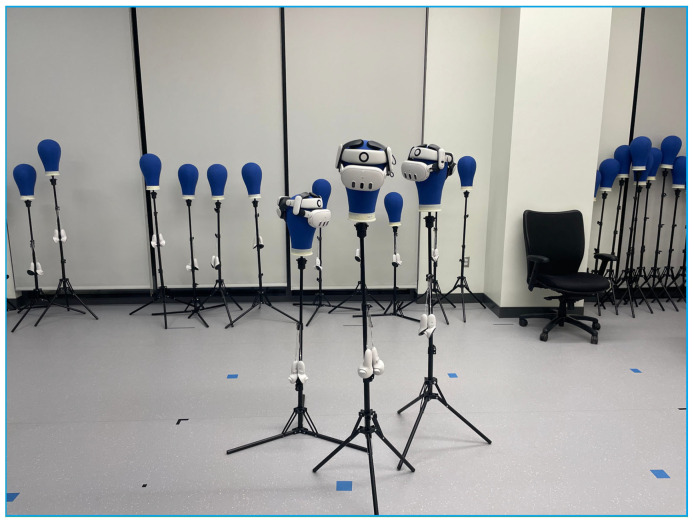
Three XR headsets in real space post calibration setup in a cluster.

Experiments were conducted using three headsets in a controlled environment, as shown in Figure 5, Figure 6, Figure 7, Figure 8, Figure 9, Figure 10, Figure 11 and Figure 12. The data from the experiments is in Appendix A. Figure 5 shows an image from the Unity editor of three connected headsets, which are localized in a shared space and ordered in a line. Figure 6 shows a view of the virtual space in Figure 5 from above. Figure 7 shows what Figure 5 and Figure 6 look like in reality. Figure 8 shows an image from above of three connected headsets which are localized in a shared space and scattered about the space in the Unity editor. Figure 9 shows what Figure 8 looks like in reality. Figure 10 shows an image from the Unity editor of three connected headsets which are localized in a shared space and in a cluster. Figure 11 shows a view of the virtual space of Figure 10 from above. Figure 12 shows what Figure 10 and Figure 11 look like in reality.

**Figure 13:**
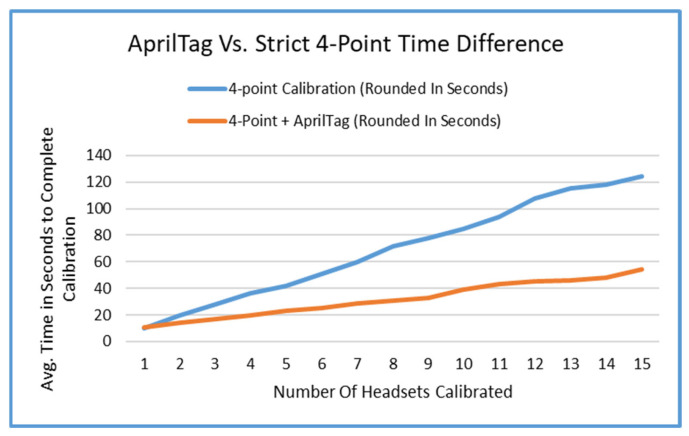
Comparative performance of AprilTag and 4-point in time.

Figure 13 shows the average difference in time it takes to localize different numbers of headsets in a scene space using AprilTags versus the four-point method. We studied the performance while trying to localize up to 15 different headsets. There were multiple trials for the two different calibrations performed for the four-point method. The first one of these was the in-place calibration. The other spanned across a space that was 18 feet by 24 feet in size. These two were averaged together, thus resulting in the baseline (blue).

### 5.1. Calibration Time Efficiency

The localization of the AprilTag method is significantly faster to calibrate when the number of headsets is scaled up. Localizing one headset takes the same amount of time, because it is essentially the same process of localization. However, once the headset’s AprilTag is positioned correctly, it is significantly faster and easier to localize the space with many headsets. Figure 13 shows the difference in time between the two methods. The four-point method data was averaged over the space of a few feet to 18 feet by 24 feet. This speed up is gained by reducing the necessity to walk to each corner of the room to localize. And ease of use is also gained from the lack of foot traffic to each point for every headset. Looking at a sheet of paper is much simpler and easier than walking across a room and bending over 15 times.

**Figure 14:**
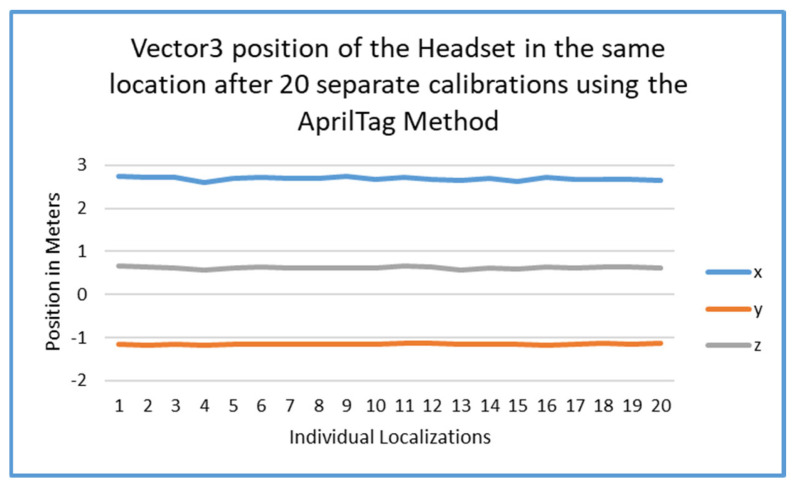
Multiple headset localizations with AprilTag.

Figure 14 displays a graph of different localizations using the same four-point calibration, but with AprilTag-based localizations. This graph shows the variance of those 20 calibrations.

**Figure 15:**
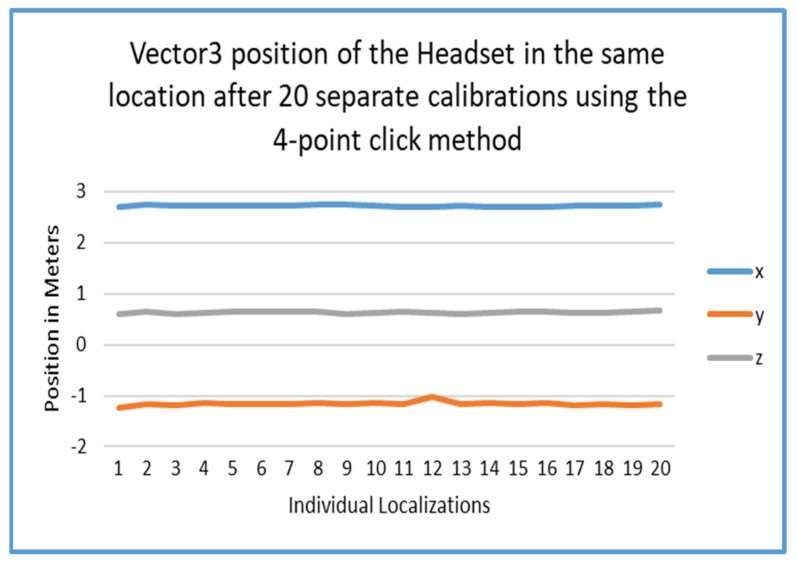
Multiple headset localizations with 4-point click.

Figure 15 depicts a graph of 20 different localizations using specific markers for the four-point click method. This graph shows the variance of those calibrations.

### 5.2. Accuracy and Error

**Figure 16:**
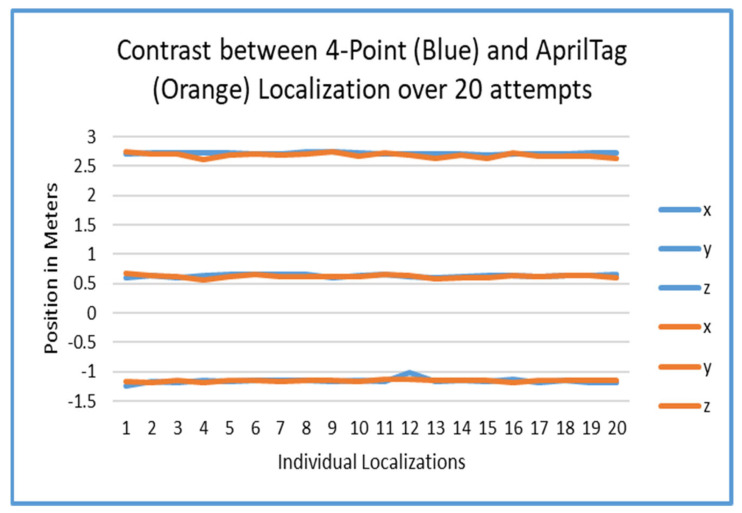
Comparative performance analysis on variance.

An analysis of the tracking error was also performed to see the difference between the two methods of localization. As seen in Figure 14, Figure 15 and Figure 16, the final localization of the players in space was very steady over the course of 20 iterations. This is very encouraging, since the AprilTag method simply reduces time and effort as opposed to the four-point method. This could only have been accomplished with smoothing algorithms which reduced rotation error in tag estimation.

In addition, visually, the headsets appear to be tracking well inside of the simulated space. As seen in Figure 5, Figure 6, Figure 7, Figure 8, Figure 9, Figure 10, Figure 11 and Figure 12, even when the headsets are moved around the test space, they can maintain their localization in both position and rotation. This is encouraging, as the headsets only require an instantaneous position and rotation from the AprilTag, as the test space itself is stationary. Thus, if the AprilTag position and rotation are correct, the localization will be correct for as long as the headset is able to maintain its tracking space.

**Figure 17:**
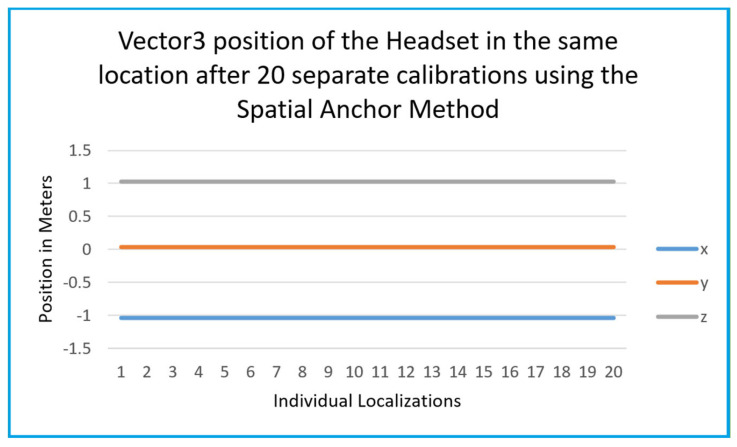
Multiple headset localizations with spatial anchors.

Data was also recorded of a headset using the spatial anchor system. As seen in Figure 17, there is a remarkable level of accuracy which spatial anchors provide. While recording data in an enclosed environment, the tracking showed minimal to no losses in pose estimation. This is to be expected, as it is a similar method to how the headset can maintain inside-out tracking. It is important to note that the use of spatial anchors is recommended when privacy is not a concern. However, as previously mentioned, to share this form of spatial information, the headsets must be connected to the internet, which may be unavailable.

## 6. Discussion

The proposed system demonstrates a viable local alternative to cloud-based spatial anchors. However, several challenges remain. We explain them in the following sections.

### 6.1. Time to Localize

Due to the need for data smoothing in tag estimation, there is a 3-second localization time that this approach requires. The data which was gathered and is represented in Figure 3 and Figure 4 show that there is a brief delay in convergence for the tags when using linear interpolation. This may be improved and could significantly reduce the time required to converge to the actual desired value.

### 6.2. Environmental Constraints

The current method may be best suited for small-to-medium sized environments. Larger spaces might require multiple AprilTags or additional anchor points to maintain high accuracy. Alternatively, other tags could be used to localize multiple divided spaces, as to reduce error in localization to a minimum. This hesitation to step into large spaces with this technology is because error still exists in the setup. So, the farther the user is from the origin of localization, the more visible the error in the system will be.

### 6.3. Dependency on Underlying Hardware

The system still utilizes the headset’s inside-out tracking. This could be a possible issue due to the nature of the AprilTag localization being instantaneous. Meaning, once the AprilTag is detected, and set, the tracking space is set in reference to the position and rotation of the tag. After the tracking space is reoriented in this way, the system simply falls back to using the native inside-out tracking of the headset. So, if tracking is lost, or tracking drifts occur, a new localization may need to be performed to reset the user in the shared space. Thankfully, it is often rare for the headsets to lose track in the short term. Most tracking losses occur when a headset is put to sleep, when a headset is turned off, or when the headset’s cameras are occluded.

Something which is a bit harder to solve is the ability to utilize the headset in large scale environments. When subjected to an environment larger than a standard room, the headsets start to have increased tracking difficulties. This is especially the case with open environments which do not have much detail. An example space which would have some difficulty is a warehouse. Often, the headsets start off well but need to be recalibrated after being used for a while. Headsets in these conditions often experience floor drift. This is often the case when the virtual environment’s floor shifts slightly upwards or downwards. This often makes virtual objects which fall to the floor unreachable since a user would need to reach past the floor to acquire said dropped object. Note that headsets are still usable in large scale environments, they are simply more prone to drift and would require re-localization more often.

A reliance on the Android Software Development Kit (SDK) for image acquisition introduces additional constraints that could be affected by future updates or restrictions on any given device [25]. This could be mitigated by either disabling updates inside of the settings menu of the headset, or, more reliably, air gapping the headset to halt any possible shadow updates which could be forced onto any given headset for security reasons. On the other hand, improvements in the hardware are equally possible. Updates could improve the fidelity of inside-out tracking as well as grant direct camera access as was seen in Meta’s v74 update [70].

### 6.4. Future Work

As future work, we plan to evaluate localization in the presence of multiple AprilTags that could improve performance and calibration accuracy through multi-lateration or multi-angulation, along similar lines as in satellite-based location evaluation services such as in GPSs. A unique challenge that would need to be addressed is that while our work was based on three-dimensional placement of the AprilTag, the GPSs must do this for receivers on a flat plane.

We could also work on placement of additional proximity sensors and similar other distance measurement sensors to aid the localization mechanism through integration with these additional sensors to improve robustness of calibration in diverse environments. Combining data from multiple sensors to create a better context could also help with internet free calibrations and localization.

Another possible line of work would be to explore the optimization of image processing algorithms. The enhancements or extensions to MARLoc [60] could be a possible research opportunity in the future for similar spaces if the reduced complexity is significant enough to run on the hardware.

As an alternative and comparable tool, quick-response (QR) Codes could also offer some promise for localization. This method is in fact now readily available through the passthrough camera API used in the camera kit [72]. In addition to this new access kit, using direct camera access to compute tags would improve the flexibility of the system to constantly view tags. In addition to that, the ability to maintain a completely virtual environment would also be gained, while still utilizing real-world localization methods with ease.

## 7. Conclusions

This paper presents a local, server-authoritative approach to spatial localization in AR/VR/XR using AprilTags. By combining a modified four-point calibration method with real-time AprilTag detection and smoothing, the system addresses shortcomings of alternative solutions. AprilTags offer faster setup times, easier setup processes, reduced overhead, equivalent fidelity, and improved privacy compared to other methods. The experimental results validate the method’s efficacy, although some limitations remain in the realms of hardware dependencies. Future research will focus on mitigating these issues by exploring additional enhancements to support larger, more complex spatial environments.

## Data Availability

All data included with the article.

## Abbreviations

The following abbreviations are used in this manuscript:

3D	Three-Dimensional
AEC	Architecture, Engineering, Construction
API	Application Programming Interface
APK	Android Package Kit
AR	Augmented Reality
CAGR	Compound Annual Growth Rate
GPS	Global Positioning System
HMD	Helmet-Mounted Display
IFL	Indoor Floor Localization
IMSA	Illinois Mathematics and Science Academy
IMU	Inertial Measurement Unit
IoT	Internet of Things
LAN	Local Area Network
MARLoc	Map-free Augmented Reality Localization
ML	Machine Learning
QR	Quick Response
SDK	Software Development Kit
SLAM	Simultaneous Localization and Mapping
UDP	User Datagram Protocol
USD	United States Dollars
VR	Virtual Reality
XR	Extended Reality

## Appendix A

The appendix contains the representative data obtained from the setup and experiments.

**Table A1:** This table is a condensed version of the data represented in Figure 3. Each second is averaged to represent the general position of the AprilTag estimation during that second.

*X*-Axis	*Y*-Axis	*Z*-Axis	Second
−0.01512	−1.19946	3.723655	0
−0.40406	−0.26484	2.29213	1
−0.97306	1.091885	0.216643	2
−0.98097	1.108534	0.191245	3
−0.97833	1.108994	0.189835	4
−0.97739	1.109204	0.191517	5
−0.97731	1.109368	0.191043	6
−0.97723	1.109649	0.190695	7
−0.97731	1.109402	0.19108	8
−0.97826	1.109221	0.191262	9
−0.97786	1.109375	0.190953	10

**Table A2:** This table is a condensed version of the data represented in Figure 4. Each second is averaged to represent the general Euler Rotation of the AprilTag estimation during that second.

Yaw	Pitch	Roll	Second
346.124	347.417	8.8998	0
308.007	311.032	264.183	1
275.458	282.186	11.392	2
275.606	281.147	12.455	3
275.217	281.618	11.343	4
275.097	283.184	9.395	5
274.971	283.536	8.809	6
275.166	283.829	8.762	7
275.099	283.144	9.446	8
275.537	284.132	8.751	9
275.370	283.432	9.599	10

**Table A3:** This table is the data represented in Figure 13. Note: There were 2 different calibrations performed for the 4-point method. One was in place. The other spanned across an 18-by-24-foot space. These two localization times were averaged together. The general time it took to localize one headset in place was about 3 to 4 s. For the larger space, it took from 12 to 16 s.

4-Point Calibration (Rounded in Seconds)	4-Point + AprilTag (Rounded in Seconds)	Title 4
10	11	1
20	14	2
28	17	3
36	20	4
42	23	5
51	25	6
60	29	7
72	31	8
78	33	9
85	39	10
94	43	11
108	45	12
115	46	13
118	48	14
128	54	15

**Table A4:** This table is the data represented in Figure 14. For each calibration, the position (*x*, *y*, and *z* values) of the player was recorded. This was performed by consistently voiding and recalibrating the headset based on a single initial calibration using the 4-point method.

*X*-Axis	*Y*-Axis	*Z*-Axis	Localization Iteration
2.749886	−1.16371	0.674176	1
2.716961	−1.18126	0.636604	2
2.715032	−1.15729	0.616984	3
2.611411	−1.18138	0.568017	4
2.696311	−1.15748	0.610536	5
2.716942	−1.15351	0.647799	6
2.68878	−1.15898	0.61798	7
2.703748	−1.14506	0.618391	8
2.737527	−1.14705	0.611675	9
2.678916	−1.16101	0.610614	10
2.727022	−1.13917	0.655564	11
2.684027	−1.13383	0.633979	12
2.639431	−1.15819	0.577344	13
2.689452	−1.15144	0.60366	14
2.634428	−1.15237	0.601827	15
2.729742	−1.18609	0.645245	16
2.662724	−1.14885	0.620524	17
2.661188	−1.14265	0.628946	18
2.67111	−1.15793	0.638476	19
2.641902	−1.14095	0.60818	20

**Table A5:** This table is the data represented in Figure 15. For each calibration, the position (*x*, *y*, and *z* values) of the player was recorded. This was performed by recalibrating the headset multiple times using the 4-point method. 4 points were marked in real space. Then, the points were marked using the controller in those specific locations in the simulator. This was performed to achieve as close to the same calibration as possible.

*X*-Axis	*Y*-Axis	*Z*-Axis	Localization Iteration
2.707758	−1.24611	0.605277	1
2.737086	−1.16327	0.645588	2
2.730683	−1.18206	0.6035	3
2.725811	−1.15339	0.631434	4
2.725961	−1.16057	0.651271	5
2.713802	−1.15669	0.649282	6
2.713802	−1.15669	0.649282	7
2.741702	−1.1515	0.650435	8
2.737857	−1.17466	0.605441	9
2.72193	−1.14643	0.627991	10
2.702918	−1.16999	0.652861	11
2.710276	−1.01463	0.622671	12
2.714176	−1.16959	0.605731	13
2.709067	−1.14114	0.626736	14
2.698106	−1.16987	0.640311	15
2.701782	−1.1346	0.637852	16
2.717204	−1.18318	0.626386	17
2.717403	−1.15774	0.631099	18
2.734392	−1.18096	0.63705	19
2.736026	−1.17749	0.659663	20

**Table A6:** This table is the data represented in Figure 17. For each calibration, the position (*x*, *y*, and *z* values) of the player was recorded. This was performed by recalibrating the headset multiple times using the spatial anchor method. A position was marked off in virtual space, and spatial anchors were positioned and saved in those locations. Then, the spatial anchors were re-loaded where they were initially marked using the headset’s spatial anchor detection system.

*X*-Axis	*Y*-Axis	*Z*-Axis	Localization Iteration
−1.0379	0.0365	1.0296	1
−1.0379	0.0365	1.0296	2
−1.0379	0.0365	1.0296	3
−1.0379	0.0365	1.0296	4
−1.0379	0.0365	1.0296	5
−1.0379	0.0365	1.0296	6
−1.0379	0.0365	1.0296	7
−1.0379	0.0365	1.0296	8
−1.0379	0.0365	1.0296	9
−1.0379	0.0365	1.0296	10
−1.0379	0.0365	1.0296	11
−1.0379	0.0365	1.0296	12
−1.0379	0.0365	1.0296	13
−1.0379	0.0365	1.0296	14
−1.0379	0.0365	1.0296	15
−1.0379	0.0365	1.0296	16
−1.0379	0.0365	1.0296	17
−1.0379	0.0365	1.0296	18
−1.0379	0.0365	1.0296	19
−1.0379	0.0365	1.0296	20
